# Code for ethical international recruitment practices: the CGFNS alliance case study

**DOI:** 10.1186/s12960-016-0127-6

**Published:** 2016-06-30

**Authors:** Franklin A. Shaffer, Mukul Bakhshi, Julia To Dutka, Janice Phillips

**Affiliations:** CGFNS International, Inc, 3600 Market Street, STE 400, Philadelphia, 19104 PA USA

**Keywords:** Code, Enforcement, Ethical, Global, Migration, Mobility, Nurses, Planning, Recruitment, Workforce, WHO code

## Abstract

Projections indicate a global workforce shortage of approximately 4.3 million across the health professions. The need to ensure an adequate supply of health workers worldwide has created a context for the increased global migration of these professionals. The global trend in the migration of health professionals has given rise to the international recruitment industry to facilitate the passage of health workers from source to destination countries. This is particularly the case in the United States, where the majority of immigrant health professionals have come by way of the recruiting industry. This industry is largely unregulated in the United States as well as in many other countries, for which voluntary codes have been used as a means to increase transparency of the recruitment process, shape professional conduct, and mitigate harm to foreign-educated health workers. The CGFNS Alliance case study presented herein describes a multi-stakeholder effort in the United States to promote ethical recruitment practices. Such codes not only complement the WHO Global Code of Practice but are necessary to maximize the impact of these global standards on local settings. This case study offers both a historical perspective and a conceptual framework for examining the multiplicity of factors affecting the migration of human resources for health. The lessons learned provide critical insights into the factors pertaining to the relevancy and effectiveness of the WHO Code from the perspectives of both source and destination countries. This study provides a conceptual model for examining the usefulness of the WHO Code as well as how best to ensure its viability, sustainability, relevancy, and effectiveness in the global environment. This case study concludes with recommendations for evolving business models that need to be in place to strengthen the effectiveness of the WHO Code in the marketplace and to ensure its impact on the international recruitment industry in advancing ethical practices. These recommendations include using effective screening mechanisms to determine health professionals’ readiness for migration as well as implementing certification processes to raise the practice standards for those directly involved in recruiting skilled workers and managing the migration flow.

## Background

Many of the issues that led to the development of the WHO Global Code of Practice on the International Recruitment of Health Personnel (WHO Code) in 2010 [1] were already being addressed in the United States by the Alliance for Ethical International Recruitment Practices through the Voluntary Code of Ethical Conduct for the Recruitment of Foreign-Educated Health Professionals to the United States (Alliance Code) launched in 2008 [2]. The size of the United States nurse workforce—representing about one-fifth of the world’s supply—and the comparatively high salaries in the United States labour market made United States healthcare employers rely heavily on the recruitment industry to ensure the supply of healthcare professionals [3, 4]. Given the increased international recruitment of nurses in the United States due to a workforce shortage in the years leading up to 2008, a multi-stakeholder task force was launched to examine the practices, mitigate the harms, and enhance the benefits of international recruitment, resulting in the creation of the Alliance Code [2]. While the WHO and Alliance Codes share similar guiding principles, they are two distinct initiatives with separate oversight and reporting requirements [1]. Thus, the present case study aims to elucidate (1) the evolution of the Alliance Code and (2) current issues relative to its implementation, as well as to describe how the two codes can work symbiotically. While a detailed discussion on the WHO Code is beyond the scope of this article, further details are available elsewhere [1].

**Fig. 1:**
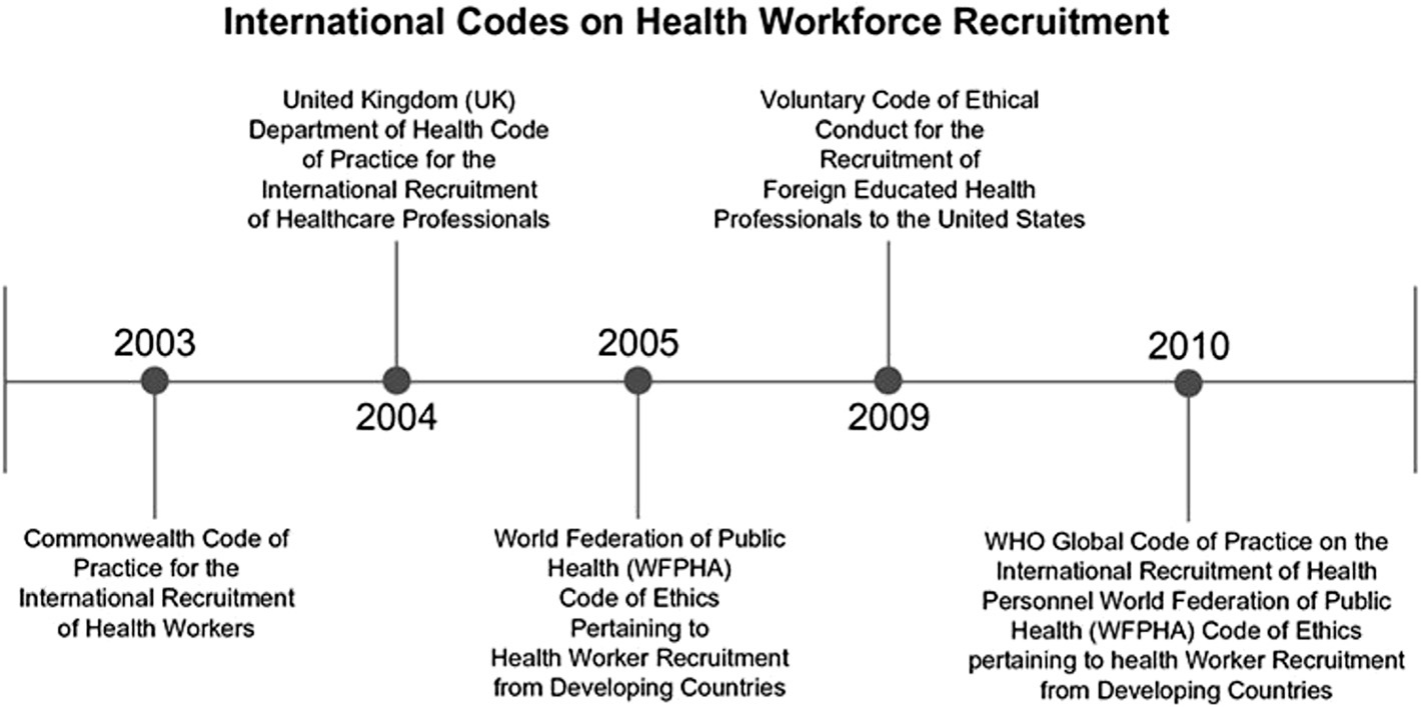
Timeline for International Codes on health workforce recruitment

With projections indicating a current global health workforce shortage of approximately 4.3 million [5, 6], the need to ensure an adequate supply of health workers worldwide is of such growing concern that WHO declared the active recruitment of healthcare workers and its related migration as one of the greatest global health threats in the 21st century [7]. Over time, numerous organizations, governments and global health advocates have attempted to regulate international recruitment by providing guidelines and specific declarations to help ensure that all health workers are recruited ethically. These guidelines and declarations were also designed to aid in reducing the recruitment of health workers from developing countries who already struggle to cope with severe health worker shortages. The Alliance Code [2] represents one of several codes adopted in individual countries to strengthen the practice of international recruitment of health personnel; Fig. 1 provides the chronology of such individual country efforts as well as that of the WHO Code.

## Case Presentation: The CGFNS Alliance case study

### Origins of the Alliance Code

Supported by the MacArthur Foundation, a 2-year, two-part effort was launched by Academy Health in 2006 to first understand the structure of the health professional labour market and institutional incentives, and to then examine the varying staffing models and industry practices.

**Fig. 2:**
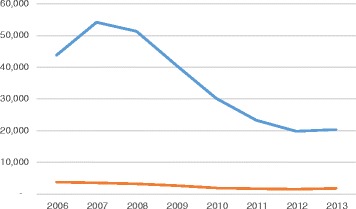
Number of foreign-educated NCLEX-RN and NCLEX-PN candidates from 2005 to 2014. Blue line, NCLEX-RN Candidates (foreign educated); Orange line, NCLEX-PN Candidates (foreign educated)

**Fig. 3:**
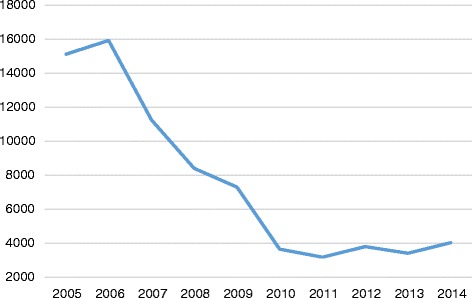
Number of *VisaScreen*® certificates issued—registered nurses and practical nurses—from 2005 to 2014

**Fig. 4:**
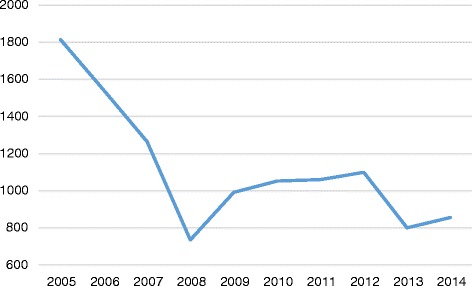
Number of *VisaScreen*® certificates issued—other professions—from 2005 to 2014

The Task Force began its work during a period of high levels of nurse migration that began declining in 2007, as demonstrated by NCLEX® data. Nurses seeking entry-to-practice in the United States are required to take the NCLEX® examinations. Figure 2 shows historical data on the number of foreign-educated nurses taking the NCLEX® for professional licensure between 2006 and 2014. The data trends provide a perspective on the pattern of nurse migration to the United States during the reported period. The significant downward trending of data reflects the onset of retrogression, a procedural delay in processing employment-based visa applications in the United States. Coupling retrogression with the worldwide financial crisis at the time, many foreign-educated health professionals found themselves unable to move forward with their desire to migrate to the United States for employment. Figure 3 shows historical data on the number of foreign-educated nurses being issued *VisaScreen®* Certificates by the CGFNS’ *VisaScreen Visa Credentials Assessment Service*® to enter the United States for employment purposes between 2005 and 2014, extracted from the CGFNS operational database. The precipitous drop starting from 2006 in the nursing sector is further corroborated by data on the other health professions for which CGFNS is authorized to perform visa screening on behalf of the federal government (Fig. 4), including nurses, medical technologists, physical therapists, occupational therapists, speech-language pathologists, audiologists, and physician assistants. However, contrary to the information provided by two different data sources at CGFNS, recruiting industry projections assumed that the upward trend would continue unabated. This was demonstrated by a 2006 CGFNS market survey of recruiters indicating that 74 % of recruiters expected to increase their international recruitment in 2007 [3]. Moreover, to capitalize on this labour flow, the number of international recruitment firms increased; in the late 1990’s, 30–40 United States-based companies were active in nurse recruiting whereas, less than a decade later, the number had ballooned, with at least 267 United States-based international recruiting firms identified by the survey [3].

In such an environment, many foreign-educated health professionals faced unfair treatment, including coercion, not being given the opportunity to review contracts, and unfair terms. Onerous contract terms and high ‘breach fee’ clauses served to trap workers in job positions with poor working conditions or benefits. Recruiters and employers often failed to meet their obligations; bait-and-switches in terms of location and staffing models were common. Finally, foreign-educated health professionals were often subjected to lower pay and less desirable assignments despite federal visa requirements often mandating that immigrant workers be paid the prevailing market wage. Indeed, the pay differential could be up to 25 % [3, 4].

With the baseline determination that (1) foreign recruitment of healthcare professionals would increase and that (2) these workers faced a host of problems due to unethical and exploitative recruitment and employment practices, the project began to develop ‘standards of practice’ to ensure ethical recruitment. A multi-stakeholder Task Force, spearheaded by Academy Health, was charged with developing these standards. The Task Force encompassed hospitals and employers, labour, nurse training and licensure organizations, foreign nurse professional associations, and recruiters, and some meetings observed by senior U.S. government officials [3]. As nurses from the Philippines, India, and Canada represent the primary sources of foreign-educated nurses to the United States, the Alliance included representatives of the Philippine Nurses Association of America, the National Association of Indian Nurses in America, and a Philippines-based recruiter to ensure a thorough understanding of the challenges faced by those considering emigration.

### The Alliance Code

The result of that effort was consensus on the Alliance Code [2]. The Alliance Code was divided into two sections. Part I established minimum standards for employers and recruiters, including commitments to adhere to applicable United States laws, to communicate transparently, and to impose reasonable and ethical contractual provisions. Provisions included adherence to applicable laws, forthright communications that specify the nature of employment and work site in detail, the ability of applicants to review contracts and not have their documents withheld, and adequate cultural and clinical training as workers transition to their life and job in the United States. Part II established aspirational best practices to protect the right to labour autonomy of foreign-educated nurses while acknowledging and mitigating the harms to source countries healthcare systems caused by the migration of nurses.

With additional funding from the MacArthur Foundation, a Stakeholder Transition Group facilitated the shift from developing the Alliance Code to implementing it in the fall of 2008. The Stakeholder Transition Group developed the strategic plan that gave rise to the Alliance for Ethical International Recruitment Practices, which developed monitoring and verification systems to assert its relevance to the issues faced by foreign-educated nurses and its effectiveness in ensuring that certified recruiters were adhering to their commitments. A pilot plan was held in 2010–2011 before the Alliance Code officially came into effect. At that time, the Alliance Code was expanded beyond foreign-educated nurses to encompass all foreign-educated health professionals [3].

The launch of the Alliance Code represented a strong multi-stakeholder commitment to protecting the interests of foreign-educated health professionals. For recruiters, it represented a willingness to believe that the imprimatur from being certified as an ethical recruiter could ultimately be a more ethical and rewarding business model by helping to differentiate their services from more exploitative competitors. While the Alliance Code was developed by stakeholders from across the health sector, its primary focus was on the recruitment industry, with the Alliance certifying and monitoring recruiters to strengthen ethical practices.

However, the financial crash and resulting global recession vastly changed the projections and market realities that had undergirded the efforts for ethical recruitment. The nurse shortage disappeared and retrogression, which delayed visas due to oversubscription, throttled the immigration of nurses. The Alliance Code provides minimal limitations on recruiter operations and provisions regarding contract transparency and adequate transition support, which are also arguably effective business practices. Nevertheless, as major recruiting firms consolidated, others felt little pressure to commit to the strictures and monitoring of the Alliance Code.

In short, there were few business incentives to join the Alliance Code or to agree to the costs imposed for failing to conduct recruitment ethically. Several years after the Alliance Code was launched, 40 % of foreign-educated health professionals reported in a study that they “perceived their wages, benefits, or shift or unit assignments to be inferior to those of their American colleagues”. Despite the publicity surrounding the Alliance Code, the advent of the WHO Code, and increased public concern around human trafficking and labour exploitation issues, half of the actively recruited foreign-educated nurses—and 68 % of those recruited by staffing agencies – were subject to some recruitment practice deemed problematic by the Alliance Code [7]. By 2014, the Alliance recognized that another paradigm was required for the Alliance Code to maximize its marketplace impact. As a result, CGFNS International, Inc., the organization responsible for credentials evaluation, acquired the Alliance to enable it to provide a better value proposition to recruiters and a bigger platform to reach stakeholders.

### The Alliance Code in the context of the WHO Code

While the Alliance Code was developed before the WHO Code, the Alliance and WHO Codes are complementary in terms of spirit, content, and objectives; both focus on ethical international recruitment in healthcare and share the fundamental goal of minimizing the harm and enhancing the benefit that cross-border migration of health professionals can engender. The two codes support each other and work symbiotically, with the WHO Code articulating global principles and a framework for international awareness and cooperation, and the Alliance Code providing detailed guidance to individuals and companies operating in the healthcare recruitment sector. As such, while the United States government’s response to WHO may cite the Alliance Code as evidence of support for ethical recruitment, this case study and the Alliance’s research are separate from the United States’ reporting requirements under the WHO Code.

The WHO Code was formally adopted by the Member States; while it speaks to non-state actors, it primarily provides a framework for countries to incorporate in their laws. It also has a wider scope than the Alliance’s Code—while the WHO Code has sections on ethical recruitment and fair treatment of migrant workers, other sections focus on international cooperation, support to developing nations, and data collection and information exchange. Further, the promulgation of the WHO Code led to further awareness by Member States. The Alliance Code represents a ‘bottom-up’ approach involving negotiations between stakeholder representatives, including recruiters, employers, nursing organizations, unions, and researchers involved in recruitment of workers to the United States. Partly because of this more limited scope, the Alliance Code provides more detailed guidance to parties on how to conduct their operations. The Alliance must communicate its Code through a variety of mechanisms, including direct outreach to recruiters and employers and social media and educational modules to inform individual migrant health professionals of their rights.

Expansion of the Alliance Code model could advance the implementation goals of the WHO Code. From a compliance perspective, the Alliance Code involves surveys of foreign-educated health professionals who use certified recruiters. A national reporting instrument every 3 years is completed by a designated governmental entity to comply with the WHO Code. As such, while the WHO Code articulates core principles involving fair treatment of migrant workers, it delegates Member States with the task of implementing policies to ensure such treatment occurs despite the labour market reality of a ready migrant workforce willing to accept jobs with lower wages or benefits or poorer working conditions than domestic workers. A regulatory approach could address these issues. Alternatively, governmental support for voluntary initiatives such as the Alliance Code could provide a more detailed framework, along with measures to ensure adherence to those protocols, to ensure that the WHO Code principles trickle down into how foreign-educated health professionals are treated by recruiters and employers. Regardless of the approach, continued pressure and attention is necessary to ensure that stakeholders adhere to their commitments. With voluntary initiatives such as the Alliance and WHO Codes, the endorsement of key principles may not translate into their actual adherence due to the lack of continued pressure from stakeholders.

## Discussion

### Introspective and prospective perspectives

Migration patterns have created challenges not only for the migrating health workers but also for source and destination countries. The recruitment industry is certainly at the centre of this global labour movement. A major challenge for source and destination countries is the establishment of workforce planning mechanisms that effectively meet human resources for health requirements. There is a growing interdependency of labour markets worldwide and thus a growing need for national and international health workforce policies. In general, countries that receive migrating health workers employ a variety of approaches to ensure that they are prepared to practice competently and safely in new, and often unfamiliar, health systems and cultures [8]; these approaches are regulated. On the other hand, the unregulated aspects of international recruitment are often left unattended since jurisdictional control over recruitment practices across borders is often neither well defined nor understood. The relevancy of the WHO Code therefore fills an unmet need by elevating this critical social discourse to the global level and by giving it the credence that it deserves. While recognizing the merits of individual regional and country efforts in establishing their respective codes, the WHO Code further acknowledges that the global nature of this work requires a platform that transcends national boundaries to achieve effectiveness.

This paper, pursued within the framework of a case study, seeks to accentuate the interplay between global responsibilities and local efforts in the United States. The case study method, as applied to the CGFNS Alliance, supports a more rigorous examination of specific issues representative of a larger corpus of matters pertaining to ethical international recruitment practices.

From this case study, the Alliance Code for ethical international recruitment as established in the United States renders some critical insights regarding its relative relevancy and continual effectiveness in achieving its stated goals.

### Lessons learned

- The multi-stakeholder approach
  - The Alliance was created using a multi-stakeholder approach to include a broad representation of different sectors of the health industry and their intersections with the recruitment industry. As such, the Alliance has provided a forum for all stakeholders to come together to work on the most pressing and sensitive issues affecting the international recruitment of healthcare professionals. This tradition of co-operation and collaboration in the Alliance has enabled it to build trust among constituent groups, including regulators, employers, recruiters, unions, professional organizations, and beyond.
- The impact of immigration policy
  - The membership of the Alliance was significantly affected by the immigration policy in the United States. As a case in point, retrogression, a procedural delay in processing employment-based visa applications, started in 2006. As documented in this case study, the blockade created by this change in immigration visa processing, coupled with the global economic downturn, has made international passage inaccessible to many foreign-educated health professionals, resulting in a drastic decrease in Alliance membership as the business for these recruitment agencies dwindled to only a handful of recruits.
- Enforcing compliance
  - The voluntary nature of the Alliance Code captures the spirit of individual self-monitoring to achieve the intended objectives of promoting transparency and fairness while reducing harm to internationally-educated healthcare professionals. The Alliance Code was largely enforced by surveying these professionals recruited by certified recruiters, but the Alliance relied on the recruiters to provide the names of the survey recipients. This rather indirect method of data collection affects the rigor of the research methodology, as well as the interpretation of findings and the probable enforcement of the Alliance Code. The feasibility for compliance enforcement given the voluntary nature of the Alliance Code will require continual monitoring and attention.
- Capacity and capability building
  - Professional growth and development has not as yet been integral to the identity of the Alliance. Programs targeted to provide for capacity and capability building of recruitment agencies would enhance their ability to perform in the international arena and ensure that their front-line employees understand the importance of ethical recruitment practices. Certification of agency staff for international recruitment work should rank high among the priorities for this evolution.
- Credential assessment of foreign-educated healthcare professionals
  - Foreign-educated healthcare professionals are generally required to undergo credential assessment to secure an employment-based visa and to meet regulatory standards for entry-to-practice in the respective professions upon entering the United States. Credential assessment is therefore central to ethical international recruitment practices in that it provides both the recruiters and those recruited with a common language in determining feasibility for migration as well as opportunities for strategic interventions to ease the passage. Looking at credential assessment from this vantage point, this evaluative process, often viewed as a barrier for migration, can in fact be an aid to ethical recruitment. Beyond serving individual health workers, CGFNS and the Alliance are in an enviable position to study how credential assessment can serve as a methodological tool to foster multilateralism and cooperation between and among source and destination countries. By making the relative alignment of different systems of education and preparation for the respective health professions more transparent, both the recruiters and the foreign-educated health professions are better informed regarding the opportunities and challenges ahead. This transparency provides a solid foundation for ethical international recruitment practices.

## Conclusions

Looking to the future, as health workers choose to work in different locations worldwide for personal or professional reasons, they bring into focus the challenges inherent in the migratory journey. They also bring into focus the necessity for continual vigilance on a code of conduct for the ethical recruitment of these professionals. This case study offers a replicable model for consideration.

Based on the continuous expansion of global labour mobility for foreign-educated health professionals, the relevance of the WHO and Alliance Codes in promoting ethical international recruitment practices is evident. Beyond the level of building awareness, these voluntary codes, however, would be difficult to sustain without a sound governance and financial and operating infrastructure to support the vision and mission inherent in individual country efforts. The development of new products and services for the industry as well as for the foreign-educated health professionals may offer conceptual and business models to strengthen the effectiveness of these Codes in the context of this labour flow.

This ethical foundation for recruitment should be fundamental to any workforce planning effort aimed to achieve human resources for health objectives. This, in turn, may pave the way for a more seamless migration of health personnel in the future.

## References

[1] World Health Organization. The WHO Global Code of Practice on the International Recruitment of Health Personnel. Geneva: WHO: 2010. http://www.who.int/hrh/migration/code/practice/en/. Accessed 28 February 2015.

[2] Alliance for Ethical International Recruitment Practices. Voluntary code of ethical conduct for the recruitment of foreign educated health professionals to the United States. Washington, DC: AEIRP; 2008. http://www.cgfnsalliance.org/about-the-codes. Accessed 28 February 2015.

[3] Pittman P, Aiken LH, Buchan J (2007). International migration of nurses: introduction. Health Serv Res.

[4] Pittman PM, Folsom AJ, Bass E (2010). U.S.-based recruitment of foreign-educated nurses: implications of an emerging industry. Am J Nurs.

[5] Crisp N, Chen L (2014). Global supply of health professionals. N Eng J Med.

[6] Taylor AL, Hwenda L, Larsen BI, Daulaire N (2011). Stemming the brain drain—a WHO global code of practice on international recruitment of health personnel. N Eng J Med.

[7] World Health Organization (2006). World health report 2006—working together for health.

[8] Nichols BL, Davis CR, Richardson DR (2011). International models of nursing. Institute of medicine, the future of nursing: leading change, advancing health.

